# Growth attenuation under saline stress is mediated by the heterotrimeric G protein complex

**DOI:** 10.1186/1471-2229-14-129

**Published:** 2014-05-12

**Authors:** Alejandro C Colaneri, Meral Tunc-Ozdemir, Jian Ping Huang, Alan M Jones

**Affiliations:** 1 Department of Biology, University of North Carolina at Chapel Hill, Chapel Hill NC, 27599, USA; 2 Department of Pharmacology, University of North Carolina at Chapel Hill, Chapel Hill, NC, 27599, USA

## Abstract

**Background:**

Plant growth is plastic, able to rapidly adjust to fluctuation in environmental conditions such as drought and salinity. Due to long-term irrigation use in agricultural systems, soil salinity is increasing; consequently crop yield is adversely affected. It is known that salt tolerance is a quantitative trait supported by genes affecting ion homeostasis, ion transport, ion compartmentalization and ion selectivity. Less is known about pathways connecting NaCl and cell proliferation and cell death. Plant growth and cell proliferation is, in part, controlled by the concerted activity of the heterotrimeric G-protein complex with glucose. Prompted by the abundance of stress-related, functional annotations of genes encoding proteins that interact with core components of the Arabidopsis heterotrimeric G protein complex (AtRGS1, AtGPA1, AGB1, and AGG), we tested the hypothesis that G proteins modulate plant growth under salt stress.

**Results:**

Na^+^ activates G signaling as quantitated by internalization of Arabidopsis Regulator of G Signaling protein 1 (AtRGS1). Despite being components of a singular signaling complex loss of the Gβ subunit (*agb1*-2 mutant) conferred accelerated senescence and aborted development in the presence of Na^+^, whereas loss of AtRGS1 (*rgs1*-2 mutant) conferred Na^+^ tolerance evident as less attenuated shoot growth and senescence. Site-directed changes in the Gα and Gβγ protein-protein interface were made to disrupt the interaction between the Gα and Gβγ subunits in order to elevate free activated Gα subunit and free Gβγ dimer at the plasma membrane. These mutations conferred sodium tolerance. Glucose in the growth media improved the survival under salt stress in Col but not in *agb1*-2 or *rgs1*-2 mutants.

**Conclusions:**

These results demonstrate a direct role for G-protein signaling in the plant growth response to salt stress. The contrasting phenotypes of *agb1*-2 and *rgs1*-2 mutants suggest that G-proteins balance growth and death under salt stress. The phenotypes of the loss-of-function mutations prompted the model that during salt stress, G activation promotes growth and attenuates senescence probably by releasing ER stress.

## Background

Three hundred million hectares are irrigated worldwide. Secondary salinization of soils has become a major undesirable consequence of this agronomic activity. With a greater need to increase crop yield on less productive land, a better knowledge of the physiological basis of salt tolerance will facilitate the engineering of salt tolerant crops needed to meet the near future food demand [1-5].

NaCl is the most soluble, widespread, and abundant of the salts in soils. As low as 40 mM NaCl generates an osmotic pressure of 0.2 MPa and this stress manifests invariably as shoot growth arrest and senescence in most glycophytes [4,6]. Tolerance to salinity is commonly reflected in plant growth, which varies as the response progresses, and each phase of the adaptation may involve different signaling pathways [7]. It is generally accepted that this response is biphasic comprised of a growth attenuating osmotic phase followed by a toxic ionic phase [4,6]. The increased concentration of cytoplasmic sodium disrupts K^+^ homeostasis, affects general trans-membrane transport, and competes with Mg^2+^ at the active site of many different enzymes, thus impairing metabolism [4,8,9] manifesting as reduced cell division/expansion and increased senescence.

Despite progress in understanding the phenomenon of plant salt-tolerance, the molecular basis for sensing and responding to extracellular Na^+^ remains controversial [10,11]. It is generally accepted that Na^+^ sensing occurs via the Salt Overly Sensitive (SOS) pathway [12]. Signaling is initiated in the cytoplasm by the SOS2/SOS3 calcium-responsive protein kinase pathway and transduced to the plasma membrane to regulate the Na^+^/H^+^ antiporter (SOS1) and reinstate ionic homeostasis. However, there is still a poor understanding about the role of the plasma membrane (PM) in sensing and signaling in response to Na^+^ [8,13].

Arguably, the best understood plasma membrane signaling pathway is mediated by the heterotrimeric G protein complex [14-21]. In Arabidopsis, this complex is comprised minimally of a Gα subunit, a Gβγ dimer and a 7-transmembrane (7TM) Regulator of G Signaling (RGS) protein (AtGPA1, AGB1, AGG, AtRGS1). Genetic evidence supports a role for this G protein complex in glucose-stimulated cell proliferation and plant growth [22-32].

In animals, a G protein-coupled receptor (GPCR) catalyzes nucleotide exchange (GTP for GDP) on the Gα subunit, leading to G activation. In plants, the Gα subunit spontaneously exchanges GDP for GTP without the requirement of a GPCR [33,34]. Therefore, to regulate G protein activity, most plants use a cell surface, 7-TM-RGS protein, the prototype being AtRGS1 [24]. AtRGS1 keeps the complex in the inactive state. Sustained activation of G signaling involves physically uncoupling AtRGS1 from the G protein complex to allow spontaneous nucleotide exchange and release of the Gβγ dimer. The cell accomplishes this uncoupling by endocytosis of AtRGS1 whereby AtRGS1 cycles through the endosome while the GTP-bound AtGPA1 remains on the plasma membrane [17]. Few G protein-interacting elements that lie downstream of the activation step are known [35-38] and none of the classic G protein targets from animals are found in plants [39].

Toward obtaining these downstream elements in plants, a G protein interactome was generated to assemble the set of plant-specific effectors using yeast complementation assays [40]. This *ab initio* assembled G-protein interactome contains 544 interactions between 434 proteins (http://bioinfolab.unl.edu/emlab/Gsignal/index.pl). Among the various biological functions assigned to the 434 G-protein interactors, the response to salt stress function is overrepresented. Heterotrimeric G-protein signaling is indirectly linked with the salt response in plants other than Arabidopsis. For example, over-expression of Gα or Gβ genes obtained from *Pisum sativum* confers increased tolerance to salt in transgenic tobacco [41]. In rice, the steady-state levels of transcripts encoding the Gα and Gγ subunits are dramatically elevated by NaCl but not KCl [42,43]. Indirect links between salt response and G-signaling can also be deduced for Arabidopsis. Ablation of *AtWNK8* kinase, a key regulator of G signaling [28,44], strengthens tolerance to salt an osmotic stress [45]. GPA1 and AGB1 mediate ABA modulation of stomata aperture during stress [19,46]. Attenuation of growth and increased cell death by drought or salt is attributed, in part, to ER stress [47,48]. In Arabidopsis, ABG1 regulates the UPR through an unknown pathway [49].

Here, we show that in response to Na^+^, AtRGS internalizes which is a robust indicator that the G-protein is activated. As predicted from our functional analysis of G-protein interactors, genetic disruption of the G-signaling system results in plants with altered adaptation to salt stress. Loss of the Gβγ dimer (AGB1) confers hypersensitivity while loss of AtRGS1 confers hyposensitivity to Na^+^. We propose a mechanism to balance growth and senescence under salt stress.

## Results

### The G protein interactome suggests a role for G proteins in saline stress

Additional file 1: Data Set S1, shows that the “response to abiotic stimulus”, “response to stress”, and “metabolic process” are the top 5 most enriched GO terms for G protein interactors. A quarter of the detected plant-G-protein interactors were annotated as abiotic stimulus responsive proteins, and half are annotated as metabolism. Additional file 1: Data Set S1 is rank-ordered by p-values, consequently the top terms represent broader general annotations with less information. We used the information contained in the resulting directed acyclic graph (DAG) [50] to systematize the selection of the most informative terms that are significantly enriched among interactors (Additional file 2: Figure S1). Table 1 shows the 22 terms found at the terminal branches of the DAG, all of them were found enriched with a corrected p-value < 0.005. By focusing on the terminal nodes, the annotations provided a clearer picture of the potential biological functions governed by G-proteins and their interactors.

**Table 1:** The G-protein interactome is enriched with proteins annotated to response to salt stress

**GO term**	**Description**	**P-value**	BH* p-value	**Enrichment**	**N**	**B**	**n**	**b**
GO:0046686	response to cadmium ion	5.48E-12	1.24E-09	4.59	27460	448	401	30
GO:0009651	response to salt stress	3.49E-11	6.43E-09	3.47	27460	750	401	38
GO:0006970	response to osmotic stress	6.62E-11	1.12E-08	3.33	27460	803	401	39
GO:0010200	response to chitin	6.53E-10	9.13E-08	4.28	27460	416	401	26
GO:0006612	protein targeting to membrane	4.73E-09	4.92E-07	4.35	27460	362	401	23
GO:0010363	regulation of plant-type hypersensitive response	2.07E-08	1.91E-06	4.18	27460	360	401	22
GO:0009416	response to light stimulus	2.64E-08	2.38E-06	2.57	27460	1120	401	42
GO:0009863	salicylic acid mediated signaling pathway	3.45E-08	3.04E-06	4.24	27460	339	401	21
GO:0009620	response to fungus	5.31E-08	3.85E-06	3.68	27460	446	401	24
GO:0006006	glucose metabolic process	7.79E-08	5.36E-06	3.49	27460	490	401	25
GO:0006096	glycolysis	5.96E-07	3.10E-05	5.21	27460	184	401	14
GO:0009409	response to cold	1.05E-06	5.12E-05	2.96	27460	602	401	26
GO:0009755	hormone-mediated signaling pathway	1.12E-06	5.41E-05	2.81	27460	683	401	28
GO:0009611	response to wounding	1.40E-06	6.51E-05	3.84	27460	321	401	18
GO:0031323	regulation of cellular metabolic process	1.82E-06	8.28E-05	1.84	27460	2347	401	63
GO:0006796	phosphate-containing compound metabolic process	1.94E-06	8.66E-05	2.14	27460	1410	401	44
GO:0050832	defense response to fungus	1.98E-06	8.73E-05	3.75	27460	329	401	18
GO:0006094	gluconeogenesis	3.63E-06	1.47E-04	5.23	27460	157	401	12
GO:0019684	photosynthesis, light reaction	4.65E-06	1.78E-04	6.28	27460	109	401	10
GO:0000097	sulfur amino acid biosynthetic process	5.78E-06	2.15E-04	3.82	27460	287	401	16
GO:0006412	translation	7.18E-06	2.60E-04	3.57	27460	326	401	17
GO:0006725	cellular aromatic compound metabolic process	9.76E-06	3.47E-04	1.66	27460	3012	401	73

*BH: multiple testing correction for p values (Benjamini and Hochberg method [51]).

The table includes GO terms found in the terminals nodes of each branch in the directed acyclic graph. These nodes contain the most specific and informative annotations that resulted significantly enriched.

Enrichment  =  (b/n) / (B/N) where N is the total number of genes associated to any GO term, B is the total number of genes associated with a specific GO term, n is the number of genes in the analyzed set, and b is the number of genes in the analyzed set associated to a particular GO term.

Table 1 reveals a combination of biotic and abiotic stress responses with central metabolic processes. This suggests that a network of G protein interactors integrates nutrient availability with stress sensing to modulate growth and survival. Proteins with roles in osmotic and salt stress responses occupied one tenth of the G-interactome, and were enriched with the highest statistical support (Additional file 2: Figure S1, Table 1).

### Na^+^ activates plant G signaling

**Figure 1:**
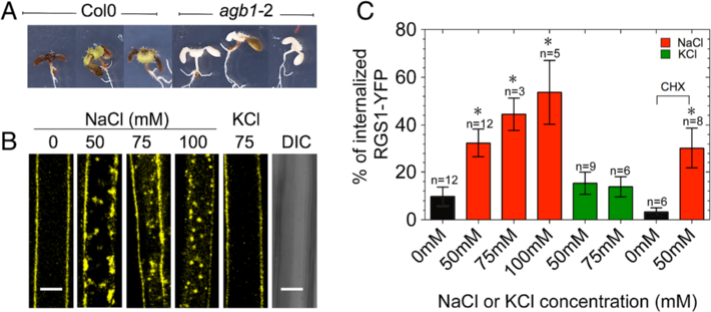
**Na**^**+ **^**triggers AtRGS1–YFP endocytosis. A)** 5-d-old *agb1*-2 and Col-0 seedlings that were grown on ¼ MS agar plates were then transplanted to ¼ MS agar plates supplemented with 200 mM NaCl. Images were captured over time, but shown are seedlings 5 d after initiation of the treatment (i.e. 10-d-old). **B)** AtRGS1–YFP endocytosis in Arabidopsis hypocotyl epidermal cells after treatment with various concentrations of NaCl or KCl for 16 h. Differential interference contrast (DIC) shows no change in cell integrity after 16 h of 100 mM NaCl treatment. The DIC is image of the same hypocotyl shown for the 100 mM NaCl treatment. **C)** AtRGS1 internalization was quantified after 16 h treatment at the indicated NaCl concentrations. CHX: seedlings were incubated with 70 μm cyclohexamide followed by water (control) or 50 mM NaCl treatment for 16 h. Error bars represent standard deviation, *n* = replicates. Pair wise comparisons between the means were performed with a T-test confidence level (CL) of 95%. All pair-wise comparisons included their respective control (no salt). *, means (treatment and control) differ significantly (p value < 0.05). Scale bars = 10 μm.

Our functional profile analysis for the G-protein interactome suggested that G proteins mediate NaCl responses. To test this hypothesis, we transplanted 5-d-old Arabidopsis seedlings of the different genotypes from ¼ MS agar plates to ¼ MS agar plates supplemented with 200 mM NaCl. Arabidopsis seedlings lacking the Gβ subunit of the heterotrimeric G protein complex (*agb1*-2) rapidly senesced (Figure 1A) compared to the wild-type. *agb1*-2 seedlings became bleached of chlorophyll while Col-0 seedlings displayed typical stress symptoms such as high levels of anthocyanin (Figure 1A) but did not bleach. This prompted the hypothesis that NaCl itself directly or indirectly activates G signaling to promote stress survival. To test activation, plants expressing AtRGS1-YFP were treated with NaCl or KCl and AtRGS1-YFP internalization was quantitated. AtRGS1 internalization is a standard reporter for G protein activation [28]. NaCl, but not KCl, initiated G signaling indicating activation is caused by Na^+^ not Cl^−^ (Figure 1B and C). Proteins visualized in the endosome after NaCl treatment had a plasma membrane origin since blocking new synthesis of protein had no effect on the subcellular location after treatment (Figure 1C).

### AtRGS and AGB1, components of the same G protein complex, have antagonistic roles in the survival of Arabidopsis to salt stress

**Figure 2:**
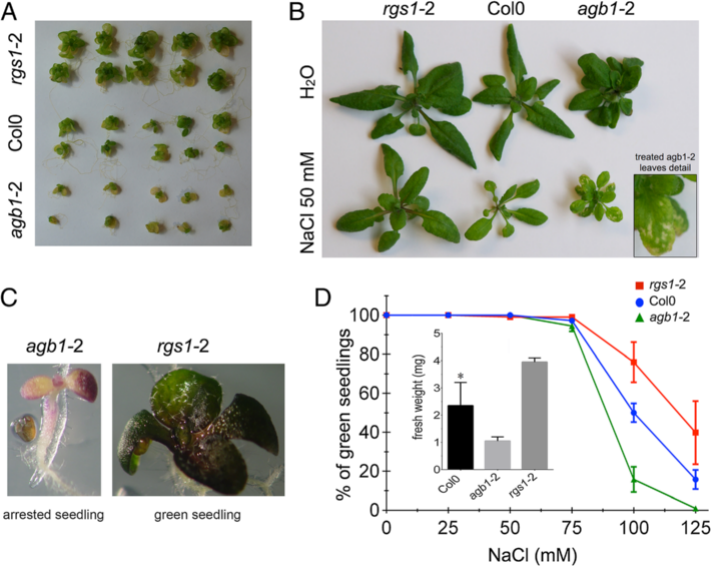
**G-protein mutants have altered responses to saline stress. A)** Col-0, *rgs1*-2 and *agb1-*2 seeds were germinated (note: they were not transplanted as in Figure 1) and seedlings grown on ¼ MS agar media supplemented with 50 mM NaCl (see Materials and Methods for this distinction in protocols). Images were captured 2 weeks after germination. **B)** Seedlings were grown on Turface Quick Dry^™^ pretreated with ¼ MS liquid media and irrigated with 50 mM NaCl solution. **C)** Examples of an arrested and a green seedling after 10 d in ¼ MS media supplemented with 125 mM NaCl **D**) Col-0, *rgs1*-2 and *agb1-*2 seeds were germinated and grown on ¼ MS agar media supplemented with different concentrations of NaCl. Green seedlings were scored 10 d after germination. Error bars represent the SEM calculated from replicate experiments- Col is statistically different from *agb1*-2 and *rgs1*-2 with a confidence level of 95% (p value = 0.0053, CL 95%).

Plants lacking AtRGS1 (*rgs1*-2) or the Gβ subunit (*agb1*-2), which is required for activation of G-signaling [28], showed clear differences in shoot growth when germinated and grown on ¼ MS agar media supplemented with NaCl (Figure 2). Attenuation of shoot growth and hastening of leaf senescence are well-characterized phenotypes displayed by plants grown under saline stress. Compared with Col-0, *agb1*-2 seedlings on NaCl were small and chlorotic. In contrast, *rgs1*-2 mutants were larger and less chlorotic than Col-0. The accelerated senescence observed in *agb1*-2 seedlings growing in NaCl-supplemented agar plates was also observed when plants were grown on soil (Figure 2B). *agb1*-2 mutants showed clear chlorotic lesions in older leaves possibly due to higher accumulation of Na^+^ in this tissue.

Arabidopsis is a glycophyte. At moderate salt concentration (e.g. 50 mM), growth is already noticeably affected and at 100 mM NaCl growth is severely inhibited. As clearly evident with the hypersensitive *agb1*-2 genotype, plant development is arrested and almost all the seedlings die at early stages (Figure 2C, 2D and Additional file 3: Figure S2). The differential sensitivities between Col0, *agb1-*2 and *rgs1-*2 were tested under different concentrations of NaCl (Figure 2D). The greatest difference among the genotypes was at 100 mM NaCl. The ability of 100 mM NaCl to arrest development was used to develop a “greening assay” to quantitate the salt-induced phenotypes of G-protein mutants (Additional file 3: Figure S2). Green seedlings that were not arrested were scored and the percent indicated for each treatment. *rgs1*-2 mutants had fewer arrested seedlings compared to Col-0, in contrast to *agb1*-2 seedlings which were almost all arrested (Additional file 3: Figure S2). These effects were not observed with the same concentration of KCl indicating that the observed phenotype is Na^+^ sodium specific (data not shown). An osmotic response is ruled out since an equal osmotic pressure applied with mannitol had no effect on shoots of the different genotypes tested (Additional file 4: Figure S3).

**Figure 3:**
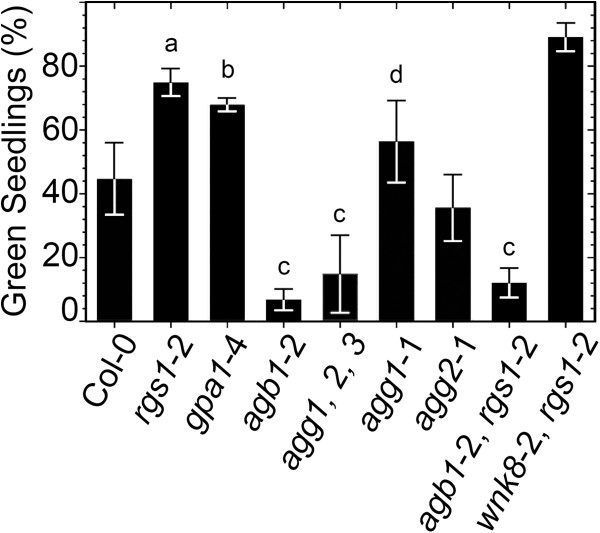
**Genetic characterization of G-protein signaling under hypersaline/hyperosmotic stress.** Arabidopsis seeds from each genotype were germinated and seedlings grown on ¼ MS agar media supplemented with 100 mM NaCl. Green seedlings were counted 10 d after germination. Error bars represent the standard deviation calculated from triplicates. Pair wise comparisons between the means were done with t-tests at a confidence level (CL) of 90% and 95%. “a” indicates that the mean for *rgs1*-2 differs from Col-0 or *rgs1*-2/*agb1*-2 double mutant (CL 95%, p-values = 0.002 and 0.005 respectively). “b” indicates *gpa1*-4 and Col 0 have different means (CL 95%, p-value = 0.02). Results are representative of three different experiments. “c” indicates that the means of these genotypes were compared with all the other genotypes and always resulted in statistical significant differences between them and all other genotypes not denoted with “c” at a CL of 95% (p value < 0.05). “d” indicates that *agg1*-1 and *agg2*-1 have different means (CL 90%, p-value = 0.051).

Seedlings lacking the Gα subunit (*gpa1*-4) behaved similarly to *rgs1* null mutants (Figure 3) consistent with AtRGS1 signaling operating through its cognate Gα subunit (t-test, p-value = 0.02, CL 95%). It also suggests that the primary signaling element is the Gβγ dimer since loss of either AtRGS1 or AtGPA1 increases the pool size of freed Gβγ dimer at the plasma membrane. As expected, when all three of the Gγ subunits are genetically deleted thus removing AGB1 from the plasma membrane, plants had the *agb1*-2 phenotype. Loss of either AGG1 or AGG2 had little or no effect suggesting functional redundancy or that AGB1 dimers comprised with AGG1 or AGG2 are not involved in the Na^+^ response. The *agb1*-2 allele was epistatic to the *rgs1*-2 allele consistent with AGB1 acting downstream of AtRGS1 (Figure 3). Like *rgs1*-2 mutants, fewer *gpa1*-4 seedlings were arrested on 100 mM NaCl compared to Col-0, however they were 30% smaller than *rgs1*-2 (t-test, p-value = 0.0005, confidence level = 95%), thus the *gpa1* “salt” phenotype is not exactly like the *rgs1* phenotype.

Endocytosis of AtRGS1 causes sustained activation of the Gα subunit and the Gβγ dimer at the plasma membrane and this process requires phosphorylation by WNK8 kinase [28]. Interestingly, mammalian homologs of plant WNK8 regulate Na^+^/K^+^ channel activity through a signaling phosphorylation pathway involving oxidative stress responsive kinases [52]. Zhang and coworkers reported that loss of WNK8 conferred salt tolerance [45]. Since WNK8 is required for AtRGS1 endocytosis, we expected that combining loss-of-function mutations in both AtRGS1 and WNK8 would be epistatic. However, loss of both AtRGS1 and WNK8 (*rgs1*-2/*wnk8*-2) conferred slightly more NaCl tolerance than the *rgs1*-2 allele alone suggesting a small additive effect (Figure 3).

### Elevating active, plasma membrane AtGPA1 subunit and AGB1/AGG dimer conferred salt tolerance

**Figure 4:**
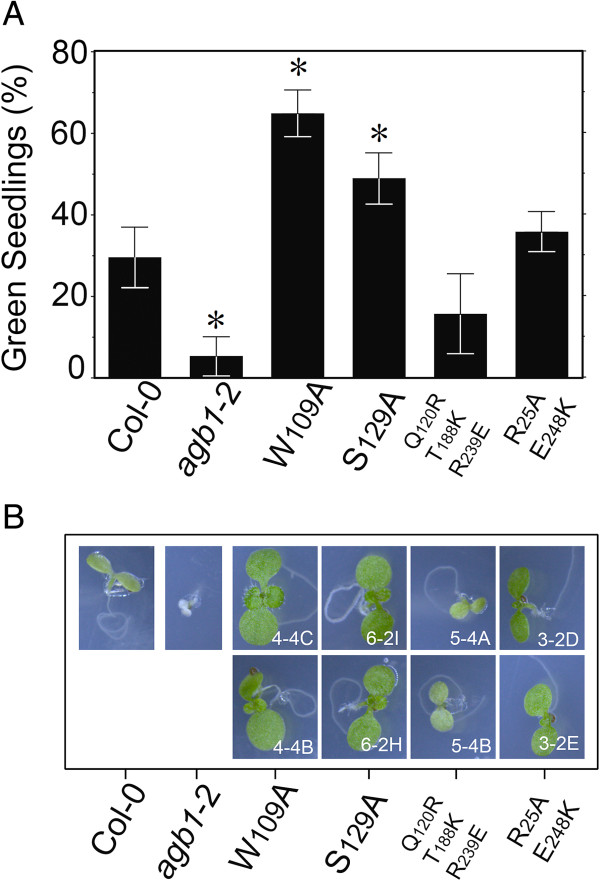
**Promoting the active state of G signaling by disfavoring heterotrimer formation confers salt tolerance.** Col-0, *agb1-*2, and Arabidopsis seedlings overexpressing (35S promoter) mutated versions of the *AGB1* gene in the *agb1*-2 background were germinated and grown in ¼ MS plates supplement with 100 mM NaCl. **A)** Green seedlings were scored 10 d after germination. Mutants are indicated with the wild-type residue position and substitution. Mutations W109A and S129A both lie in the Gα:Gβγ protein interface and thus shift the equilibrium away from heterotrimer formation to active Gα subunit and Gβγ dimer. Data is expressed as the mean of 3 replicates with 36 seedlings per genotype per plate; error bars = SD. Pair wise comparisons between the means of Col 0 plants complemented with mutant variants of the *AGB1* genes were performed with a single-tailed T-test. *, means comparison show up as statistically significant. The *agb1*-2 null mutation conferred reduced number of green seedling (CL = 99%, p value 0.0009). Plants complemented with the W109A or S129A variants of the *AGB1* gene produced higher number of green seedlings, (CL = 99%, p value 0.0042 and CL = 90%, p value 0.07), respectively). **B)** Images of seedling were taken at the end of the treatment. Two lines for *agb1*-2 seedling complemented with mutated AGB1 are shown.

To test whether AtRGS1 operates through the Gα subunit to regulate the activity of the Gβγ dimer, we used two point mutations that independently disrupt binding between AtGPA1 and AGB1 and consequently increase the pool of activated G proteins at the plasma membrane [53]. Disrupting heterotrimer formation is expected to increase the pool of active Gα and Gβγ subunits without disrupting AtRGS1 function. Mutant AGB1 proteins were expressed in the *agb1*-2 null background and at least two independent lines were characterized. Both W_109_ and S_129_ residues lie within the Gα-Gβ protein interface and mutation of these residues to alanine prevents Gα binding to Gβγ without disruption of the plasma membrane localization [53]. Reduced heterotrimer formation means an increase in activated Gα subunit and Gβγ dimer at the plasma membrane. Mutations in these residues confer NaCl tolerance (Figure 4). The positive control was a set of mutations on the surface located outside the Gα-Gβ surface of interaction (R_25_A, E_248_K double). This mutant AGB1 rescued the *agb1*-2 null mutant to wild type levels of tolerance. The negative control was a set of mutations in a surface patch (Q_120_R,T_188_K, R_239_E triple mutant) known to be involved in many cellular responses [53]. This mutant AGB1 did not rescue the *agb1*-2 salt-sensitive phenotype.

### G signaling mediates glucose-induced tolerance to NaCl

**Figure 5:**
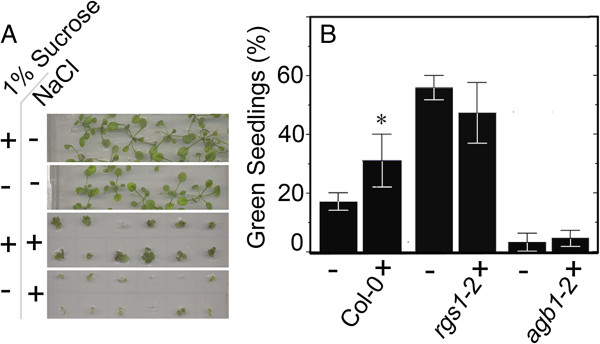
**Sugar ameliorates the salt stress. A)** Col-0, seeds were germinated and seedlings grown on 1/4 MS agar media supplemented with or without 100 mM NaCl and with or without 1% sucrose. Seedlings shown are 3 weeks old. **B)** Col-0, *rgs1*-2 and *agb1-*2 seed were germinated and seedlings grown on 1/4 MS agar media supplemented with 100 mM NaCl plus (+) or minus (−) 0.5% glucose. Green seedlings were scored 10 d after germination. Error bar represents the STDev from 3 replicates. Each genotype treated with glucose was compared to its no glucose control. *means statistically different with a P value = 0.01.

A regulatory signaling network integrating environmental cues with nutritional status may play a key role in shunting energy from developmental-linked biosynthetic metabolism into metabolic pathways aimed at boosting stress tolerance [54]. Since both AtRGS1 and AGB1 are part of a sugar-dependent signaling pathway and corresponding mutants have altered responsiveness to NaCl, we tested if sugar sensing was a factor of the NaCl response. Sucrose (Figure 5A) and glucose (Figure 5B, p value = 0.064, CL 90%), improved salt tolerance for Col-0 seedlings. Glucose had no effect on salt tolerance for *rgs1-2* or *agb1*-2 mutants (t-test, p value = 0.27 and p value = 0.49, CL 90%) at the tested concentration.

## Discussion

Geng et al. [7] elegantly showed that the reaction of a root to salt is complex and has dynamics in both temporal and spatial dimensions. Upon an initial shock to applied salt, the root growth rate dramatically decreases within the first few hours (designated the stop phase) followed by a slow constant growth rate over the next few hours (quiescent phase). During the next ~10 hours, growth recovers (recovery phase) albeit not to the full rate of the control roots and then growth reaches a new steady-state rate (homeostasis phase). This implies a complex and dynamic regulatory system. Indeed, Geng, et al. [7] showed that throughout this timeline different transcriptional programs begin and end in a tissue-specific context.

Given the delay in activation (Figure 1), G proteins are most likely to be involved in the recovery phase but the mechanism is unclear and at this juncture, we can only speculate based on the observations that we and others report. In many plants, the 7TM-RGS protein holds the Gα subunit loaded with GDP in an inactive state, which favors the formation of the inactive heterotrimer (Gα:Gβγ). Because plant Gα subunits spontaneously exchange GDP for GTP, in the absence of the 7TM-RGS, the Gα subunit is GTP bound (i.e. activated) and the Gβγ dimer is freed. Sustained activation of G signaling involves physically uncoupling 7TM-RGS from the G protein complex to allow spontaneous nucleotide exchange and release of the Gβγ dimer. The cell accomplishes this physical uncoupling by endocytosis of the 7TM-RGS whereby it cycles through the endosome while the GTP-bound Gα subunit remains on the plasma membrane [17]. It is abundantly clear that endocytosis of AtRGS1 increases the active G protein pool [44]. Upon activation, the GTP-bound Gα subunit releases the Gβγ dimer enabling both G protein components to interact with cellular targets. The Gα subunit is a positive modulator of cell proliferation in Arabidopsis [22,27] and the Gβγ dimer, among other roles it plays, operates in the UPR, which is important for cell survival during ER stress [49,55]. Salt induces the UPR in which case the UPR alleviates NaCl stress by increasing ER-associated protein degradation [56]. Failure to do so triggers salt-associated-cell-death [47]. AGB1 plays a positive role in the UPR response [49], since three tested loss-of-function *agb1* mutants showed hypersensitivity to tunicamycin (note that an earlier report by Wang et al. [55] describing the opposite *agb1* UPR phenotype could not be reproduced) [49].

The genetic data here support this biological context. In the *rgs1*-2 mutant, two important activities are increased at the plasma membrane: a proliferative factor (GTP-bound AtGPA1) and an ER-stress reliever factor (unsequestered AGB1). Consequently, mutations that promote active Gα subunit and Gβγ dimers confer sustained growth and tolerance to NaCl compared to WT. Consistent with this idea, disruption of the heterotrimer in a way that promotes active AtGPA1 and AGB1 without the loss of AtRGS1 also conferred tolerance to NaCl compared to Col-0 (Figure 4). The *gpa1*-4 phenotype is also consistent with this conclusion; in the absence of AtGPA1, plants have more activated AGB1 at the plasma membrane as for loss of AtRGS1, therefore these mutants are less sensitive to the stress. However, *gpa1* mutants lack the cell proliferation function, therefore they do not behave phenotypically exactly like *rgs1* mutants (Figure 3). In contrast, *agb1*-2 and *agb1*-2/ *rgs1*-2 double mutants lack Gβ thus making these seedlings highly sensitive to the ER-stress imposed by NaCl.

While it is possible that AtRGS1 senses Na^+^, we do not favor this view since the NaCl effect (Figure 1B) is slow, thus activation may be indirect through an increase in glucose by regulation of glucose metabolism enzymes leading to increased sugar levels. In fact, it was clearly demonstrated that NaCl increases sugar levels in root [57] and leaf cells [58]. This also explains the ameliorative effect of applied glucose on NaCl responsiveness (Figure 5).

## Conclusions

Plant tolerance to NaCl, in particular the recovery phase, involves the plasma membrane G protein-mediated glucose-signaling pathway. The mechanism for survival to salt stress requires G protein activation by releasing freed Gα subunits and Gβγ dimers. The discovery here of sodium-induced activation of G signaling via AtRGS1 endocytosis, whether or not direct or indirect through increased glucose levels, raises further complexity involving feedback loops that will need to be addressed.

## Methods

### Plant material and growth conditions

All plants were the Col-0 ecotype: *rgs1*-2 [24]; *agb1*-2 [25]; *gpa1*-4 [59]; *wnk8*-2 [28]; *agg1*-1 [60]; *agg2*-1 [61]; *agg3*-1 [62]; and the series of point mutations on AGB1 [53]. The quality of seed stock was a major factor for variability during the NaCl greening assays. All seeds were harvested from plants grown together under identical conditions. To eliminate crowding effects seeds were plated on a grid 1 cm apart. Seeds were surface sterilized with 70% ethanol solution for 10 min, and 95% ethanol solution stratified on plates at 4°C, 48 h. Media was ¼ Murashige and Skoog (Calsson Labs, Cat# MSP01) and 1.5% phytoagar (RPI Corp. Cat # A20300-1000.0), supplemented or not with NaCl, KCl, mannitol, and/or sucrose. Plates were moved to a growth chamber under constant light conditions (21°C, 60 μmole m^−2^ s^−1^). Differences among genotypes in the time of the onset of yellowing were greatest at day 10 under these conditions.It is important to note that germinating and growing these genotypes on media containing NaCl vs. transferring seedlings germinated and grown first on media lacking NaCl to media containing NaCl affects the phenotypic outcome (c.f. Figures 1A and 2A).

### Accessions

*AtRGS1*, At3G26090; *AtGPA1,* At2G26300; *AGB1*, At4G34460; *ATWNK8*, At5G41990; *AGG1*, At3G63420; *AGG2*, At3G22942; *AGG3*, At5G20635; *WNK8*, At5g41990.

### Salt tolerance in soil

Seeds were germinated on ¼ MS agar media for 7 d. Seedlings were transplanted to pots containing Turface Quick Dry™ (Turface Athletics, Buffalo Grove, IL) presoaked with ¼ MS liquid media. Plants were grown for 5 d. Healthy looking plants of each genotype were selected as control or treatment. Plants under NaCl treatment were kept under constant irrigation with 50 mM NaCl solution. Water was used as the control.

### Fresh weight determination

Seedling shoots were detached from the roots and weighed on an analytical balance. When shoots were harvested and weighed in batches, they were kept during this period in a 100% humidity chamber until reading could be taken.

### AtRGS1 internalization

Columbia-0 (Col-0) seedlings stably expressing AtRGS1-YFP fusion protein under the control of 35S cauliflower mosaic virus promoter were first grown for 5 d at dark in 1/4MS liquid media without glucose. Then media was replaced by NaCl or KCl solutions at different concentrations or just water as a control. After 16 hs of treatment in dark seedlings were observed under the microscope. Vertical optical sections (i.e. Z stacks acquired) of hypocotyl epidermal cells of dark-grown seedlings located approximately 3–4 mm from the cotyledon were captured using a Zeiss LSM710 confocal laser scanning microscope equipped with a C-Apochromat × 40 NA = 1.20 water immersion objective. YFP was excited using the 514-nm line from an argon laser and its respective emission was detected at 526–569 nm by a photomultiplier detector. The images were analyzed by using the software Image J [63] as described by Urano et al. [28]. In brief, randomly selected hypocotyl images from three or four whole *Z*-section image stacks of 3 to 5 independent experiments were selected for quantification. Fluorescence signals were subject to a minimum cut-off and the intensity was measured and subtracted from the total hypocotyl fluorescence. Statistical comparison of mean fluorescence signal was performed using Student t-test.

### Statistical analysis

#### Gene ontology (GO) enrichment analysis

Data was obtained from a G protein interactome generated with a yeast complementation assays revealing the set of G protein plant-specific effectors [40]. This interactome is of high quality because the database was constructed of multiple screens of 9 cDNA libraries using wildtype and mutant forms of G protein baits. The G-protein interactome contains at least 544 interactions between at least 434 proteins (http://bioinfolab.unl.edu/emlab/Gsignal/index.pl). This database was analyzed for gene functional annotation using Gene Ontology enRIchment anaLysis and visuaLizAtion tool (GOrilla) [64]. The DAG generated by this tool systematically establishes thresholds based on the structure of the results to reach informative GO terms. To build a background list, all the AGI codes representing the Arabidopsis genes were obtained from the TAIR web site. AGI codes were converted to official gene symbols using the gene ID conversion tool at DAVID Bioinformatics Resources 6.7 [65].

#### Pair-wise comparison of means

Unpaired Student t-test (two tailed) at a confidence level of 90% or 95% were performed with GraphPad Prism version 6.00 for windows (GraphPad Software, San Diego California USA, http://www.graphpad.com).

## Abbreviations

7TM: AGB1seven transmembrane; AtGPA1: Arabidopsis Gα subunit 1; AGB1: Arabidopsis Gβ subunit 1; AtRGS1: Arabidopsis regulator of G signaling protein 1; CL: Confidence level; ER: Endoplasmic reticulum; GPCR: G-protein-coupled receptor; mPa: Mega pascals; RGS: Regulator of G Signaling; UPR: Unfolded protein response; WNK: With no lysine kinase.

## Competing interests

The authors declare that they have no competing interests.

## Authors’ contributions

AC designed experiments characterizing the salt responsiveness of the genotypes described. AC also analyzed the G protein interactome for associations in annotations. MT-O designed and performed experiments testing salt-induced AtRGS1 endocytosis. J-PH assisted AC in some experiments and was involved in experimental design. AMJ and AC wrote the manuscript. AMJ managed the project. All authors read and approved the final manuscript.

## Supplementary Material

Additional file 1 **Data Set S1.** GO enrichment analysis results (excel file). This data set contains the functional enrichment analysis results obtained with the list of genes encoding G-protein interactors [40]. A description of the column headers in Data Sets S1 is provided at the bottom of the spreadsheet. Click here for file

Additional file 2: Figure S1 Directed acyclic graph (DAG) depicting the functional profile associated with the G-protein interactome. The picture is a simplified illustration of the entire dataset provided in Additional file 1: Data Set S1. Color code bar is the heat map reflecting the statistical support for each enriched GO term with corresponding color. Click here for file

Additional file 3: Figure S2 Complete data acquisition for 3 replicate experiments. All the NaCl green seedling assays were done according to the format shown here. **A)** Col0, *rgs1*-2 and *agb1-*2 sterilized seed were sown on squared plates with 1-cm grid (1 seed per square). Plates contained ¼ MS salts supplemented with 100 mM NaCl, or ¼ MS salts alone for controls (panel C). Seeds were stratified on plates at 4°C, 48 h. Seeds were germinated and grown in constant light conditions (60 μmole m^−2^ s^−1^) at 21°C. **B)** Green seedlings were scored 10 d after germination. The onset of yellowing (senescent seedlings vs. green tolerant seedlings) varied ± 2 d from experiment to experiment. Three replicates of genotypes, treatments and experiments were conducted. Error bars represent standard deviation of triplicates. **C and D)** Typical results found for seedlings (Col-0, *rgs1*-2 and *agb1-*2 ) after 10 d on control plates (1/4 MS, 0.8% agar). Click here for file

Additional file 4: Figure S3 Mannitol does not evoke the NaCl phenotypes in the tested genotypes. Experiments were performed as described for Figure S2 but the media was supplemented with 200 mM mannitol instead of NaCl. Click here for file
