# Supplementary material for: Growth attenuation under saline stress is mediated by the heterotrimeric G protein complex
Source: BMC Plant Biol. 2014 May 12;14:129. doi: 10.1186/1471-2229-14-129 (PMC4061919; doi:10.1186/1471-2229-14-129)
Supplement: Additional file 2: Figure S1 — Directed acyclic graph (DAG) depicting the functional profile associated with the G-protein interactome. The picture is a simplified illustration of the entire dataset provided in Additional file 1: Data Set S1. Color code bar is the heat map reflecting the statistical support for each enriched GO term with corresponding color. [file 1471-2229-14-129-S2.pdf]

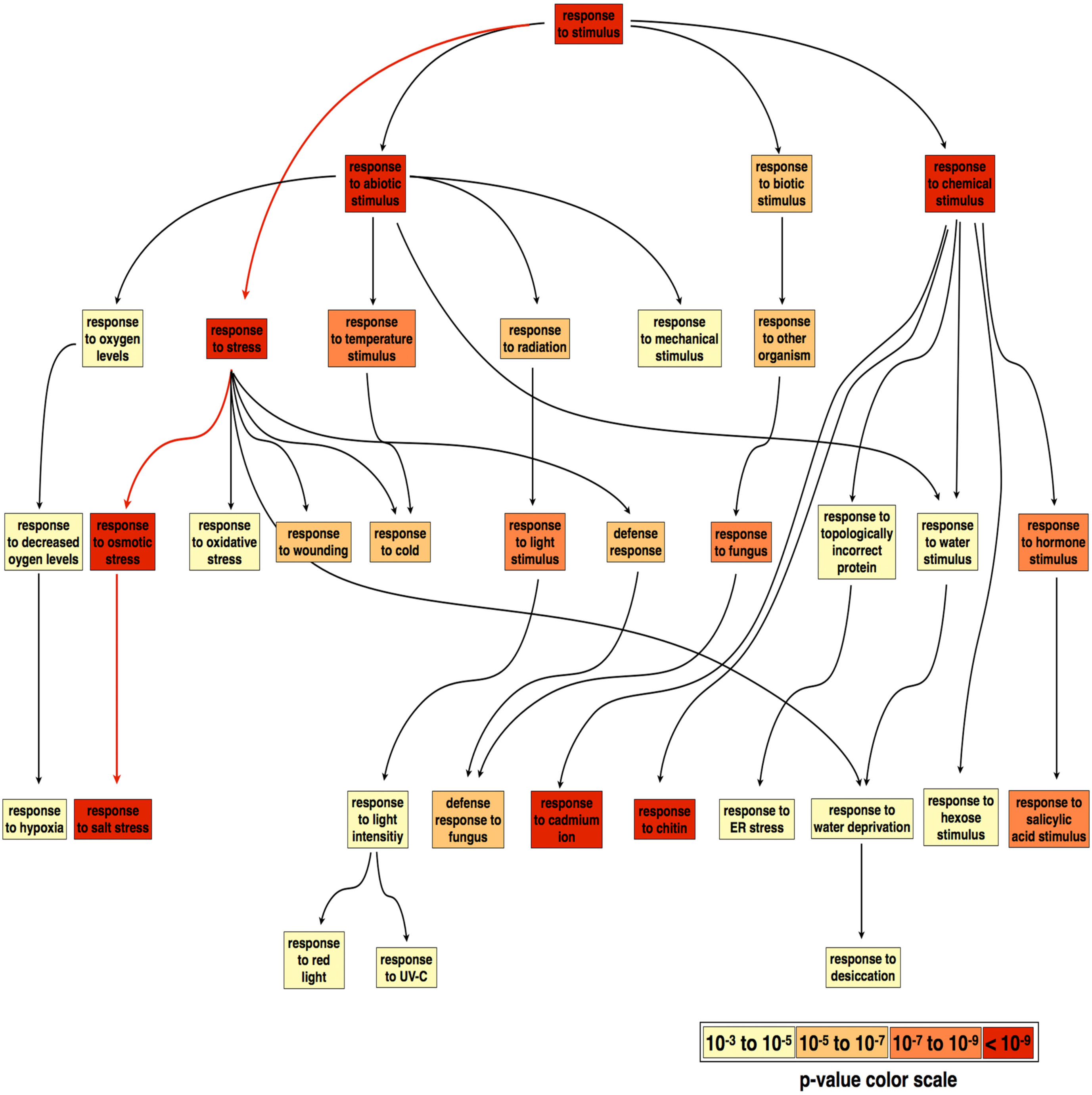

Figure S1 Directed acyclic graph (DAG) depicting the functional profile associated with the G-protein interactome. The picture is a simplified illustration of the entire dataset provided in Data Set S1. Color code bar is the heat map reflecting the statistical support for each enriched GO term with corresponding color.
