# Supplementary material for: Growth attenuation under saline stress is mediated by the heterotrimeric G protein complex
Source: BMC Plant Biol. 2014 May 12;14:129. doi: 10.1186/1471-2229-14-129 (PMC4061919; doi:10.1186/1471-2229-14-129)
Supplement: Additional file 3: Figure S2 — Complete data acquisition for 3 replicate experiments. All the NaCl green seedling assays were done according to the format shown here. A) Col0, rgs1-2 and agb1-2 sterilized seed were sown on squared plates with 1-cm grid (1 seed per square). Plates contained ¼ MS salts supplemented with 100 mM NaCl, or ¼ MS salts alone for controls (panel C). Seeds were stratified on plates at 4°C, 48 h. Seeds were germinated and grown in constant light conditions (60 μmole m−2 s−1) at 21°C. B) Green seedlings were scored 10 d after germination. The onset of yellowing (senescent seedlings vs. green tolerant seedlings) varied ± 2 d from experiment to experiment. Three replicates of genotypes, treatments and experiments were conducted. Error bars represent standard deviation of triplicates. C and D) Typical results found for seedlings (Col-0, rgs1-2 and agb1-2 ) after 10 d on control plates (1/4 MS, 0.8% agar). [file 1471-2229-14-129-S3.pdf]

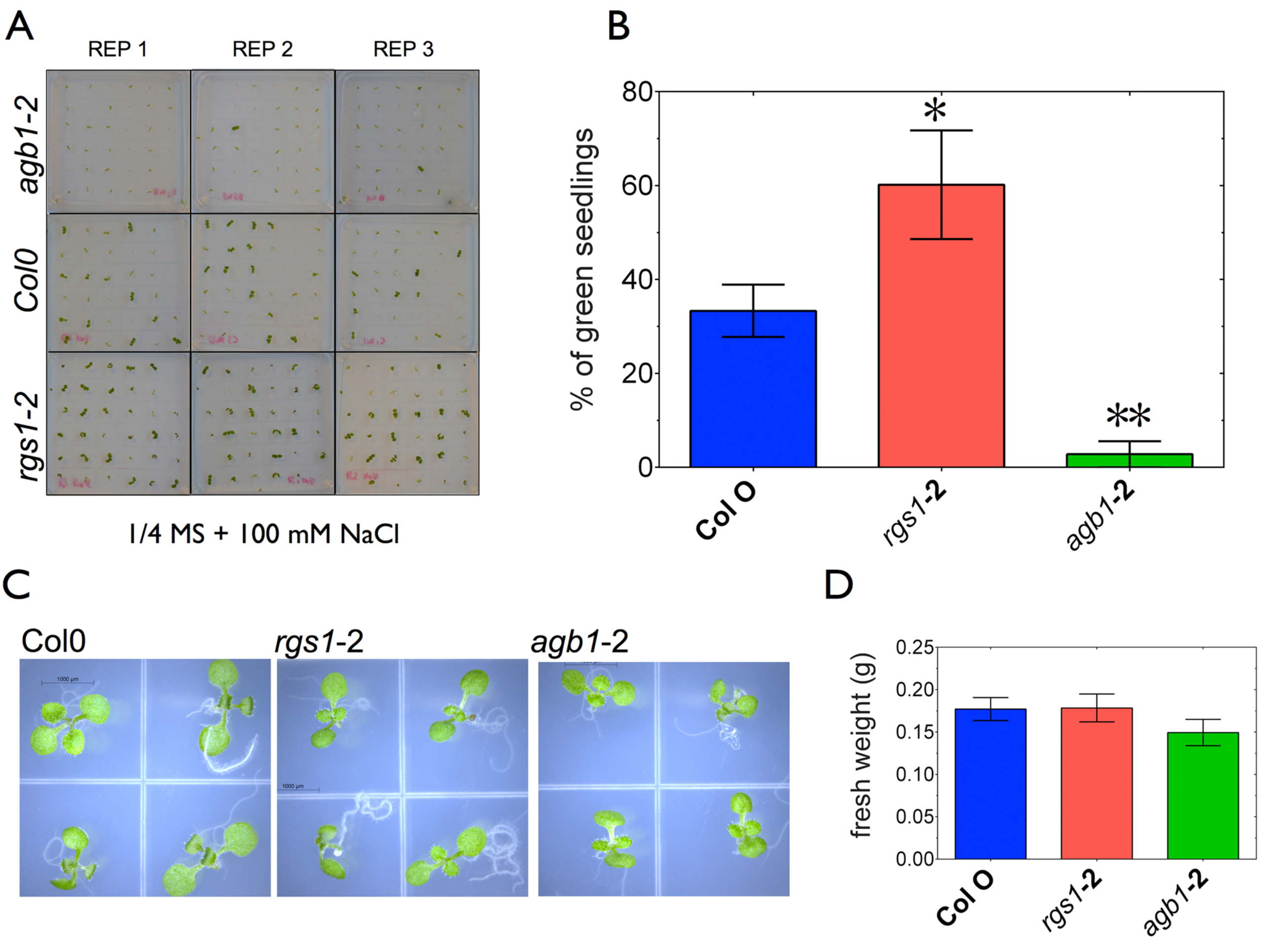


**Figure S2 Complete data acquisition for 3 replicate experiments**. All the NaCl green seedling assays were done according to the format shown here. **A)** Col0, *rgs1*-2 and *agb1*-2 sterilized seed were sown on squared plates with 1-cm grid (1 seed per square). Plates contained ¼ MS salts supplemented with 100 mM NaCl, or ¼ MS salts alone for controls (panel C). Seeds were stratified on plates at 4$^{o}$ C, 48 h. Seeds were germinated and grown in constant light conditions (60 μmole $m^{-2}$ $s^{-1}$) at 21$^{o}$C. **B)** Green seedlings were scored 10 d after germination. The onset of yellowing (senescent seedlings vs. green tolerant seedlings) varied ± 2 d from experiment to experiment. Three replicates of genotypes, treatments and experiments were conducted. Error bars represent standard deviation of triplicates. **C and D)** Typical results found for seedlings (Col-0, *rgs1*-2 and *agb1*-2 ) after 10 d on control plates (1/4 MS, 0.8% agar).
