# Supplementary material for: Growth attenuation under saline stress is mediated by the heterotrimeric G protein complex
Source: BMC Plant Biol. 2014 May 12;14:129. doi: 10.1186/1471-2229-14-129 (PMC4061919; doi:10.1186/1471-2229-14-129)
Supplement: Additional file 4: Figure S3 — Mannitol does not evoke the NaCl phenotypes in the tested genotypes. Experiments were performed as described for Figure S2 but the media was supplemented with 200 mM mannitol instead of NaCl. [file 1471-2229-14-129-S4.pdf]

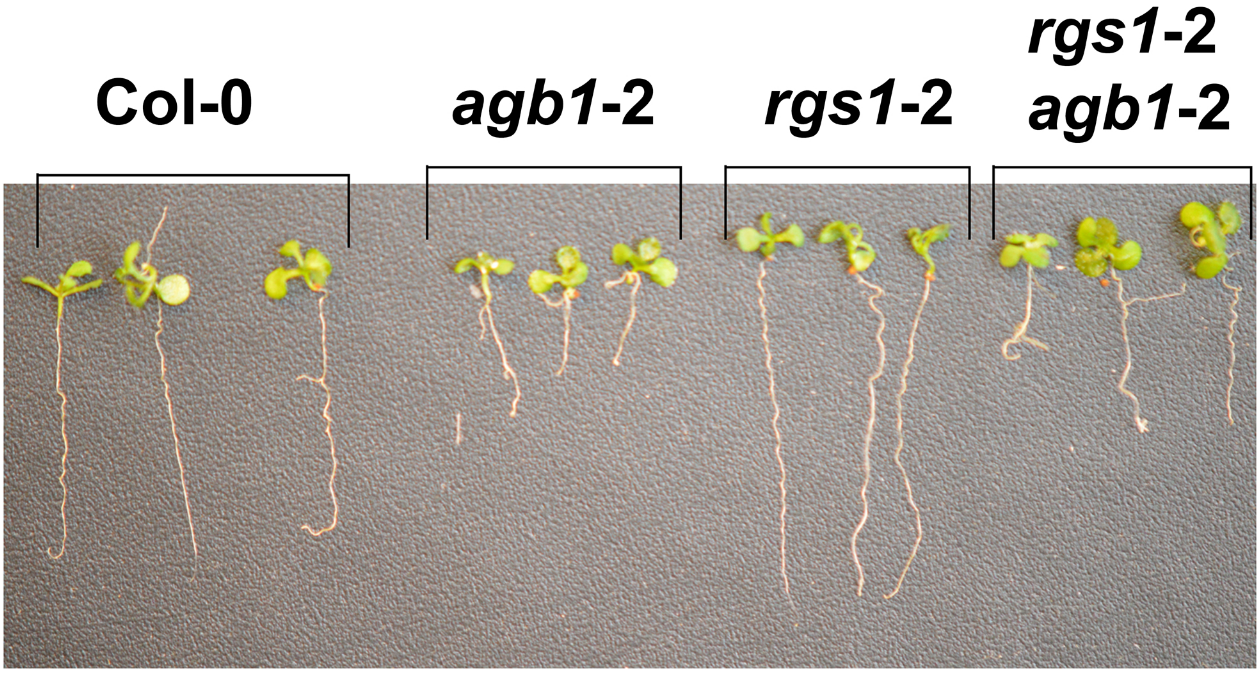

Figure S3 Mannitol does not evoke the NaCl phenotypes in the tested genotypes. Experiments were performed as described for Figure S2 but the media was supplemented with 200 mM mannitol instead of NaCl.
